# Pilot study: Post-surgical infections could be related with lack of sharpness in surgical tools

**DOI:** 10.1371/journal.pone.0261322

**Published:** 2022-02-02

**Authors:** David E. White, Jim Bartley, Christopher Whittington, Lorenzo Garcia, Kaushik Chand, Celine Turangi

**Affiliations:** 1 BioDesign Lab, School of Engineering, Computer and Mathematical Sciences, Auckland University of Technology, Auckland, New Zealand; 2 Department of Surgery, University of Auckland, Auckland, New Zealand; Universiti Teknologi Brunei, BRUNEI DARUSSALAM

## Abstract

Despite rigorous sterilization protocols placed in surgical procedures, there is demonstrated evidence that show patients contract infections while hospitalized. This study aims to investigate the presence of biological materials in osteotome surgical tools after sterilization processes, determine the relationship between lack of sharpness and cross-contamination, and evaluate the influence of materials surface coating as a potential contamination preventive. Three commercially available osteotomes with different surface coatings were studied and submitted to a procedure of bone-cutting cycles. After use, each was sterilized and examined under SEM and EDS. Bone contaminants were detected in each osteotome although the PVD coated osteotome demonstrated significantly less contamination than either the as-supplied or electroless nickel coated one. According to the results, there is an association between blade sharpness and post-sterilization bone contamination. These findings suggest either disposable osteotomes should be used in surgical procedures, or an effective sharpen process should both be established and monitored to minimise post-operative infections.

## Introduction

Several studies [1–4] has shown that patients have contracted infections while hospitalized. Despite rigorous sterilization protocols being in place, some of these deaths may have been caused by contaminated reusable surgical instruments. Post-surgical infections have resulted in a doubling of rehospitalization rates which caused a near 300% increase in healthcare costs as well as a decrease in the quality of patient life [5].

The osteotome is a surgical tool commonly used to chip, cut, and sculpt bone during various surgical procedures, such us orthopaedic, plastic, and dental surgeries. This tool resembles a chisel, beveled on both sides of the cutting edge and is like many bone-cutting surgical tools usually manufactured from either heat treated 440 Martensite, 316 Austenite, or 420 Martensitic Stainless Steel. These ductile materials allow surgical tools to withstand impact forces without fracturing. They also possess excellent properties of corrosion resistance, biocompatibility, and cost-effectiveness. Osteotomies are re-used in surgical procedures however lack of regular maintenance can lead to a dull or damaged cutting edge forming [6].

Previous study has shown degradation in osteotome cutting performance occurs after very little use [6]. We hypothesise that surface defects, due to wear along the osteotome cutting edge, could harbour pathogens that remain after post-surgery cleaning and sterilization routines, potentially exposing subsequent patients to cross-contamination and increasing the risk of post-operative infections [7]. Regardless of the type of sterilization process used, there is a risk of trapped pathogens surviving the sterilization process if surface irregularities along the cutting edge are present [8]. We found there is little work done investigating the permanence of biological material in unsharpened surgical tools after re-use.

The goal of this study is to determine if there is any relationship between lack sharpness and cross-contamination in osteotomes, and to investigate the influence of surface coating as contamination preventive.

## Materials and methods

This study utilized three identical and new commercially available osteotomes (RU 5331–30, Rudolf MedicalGmbH, Fridingen, Germany). One osteotome was electroless nickel coated to a thickness of 8μm, another was TiN coated using physical vapor deposition (PVD) to a thickness of 2μm, and the remaining one was left in the as-supplied state. Wear resistant electroless nickel and TiN coatings were each applied at the minimum viable thickness offered by their respective coating processes.

Each osteotome underwent a series of four bone cutting cycles. Bone cutting entailed holding the osteotome at an inclination with the aid of a cutting jig that replicated the procedure commonly used during surgery. Each bone cutting cycle removed a 40-mm length of cuticle bone to a depth of 5 mm along the axial direction of the freshly excised adult bovine femur. The same bone sample was utilized to ensure consistency of bone strength between each test following the same procedure described in previous work [6]. Upon completion of all four bone cutting cycles, each osteotome was then thoroughly cleaned and sterilized (described later) before a small 5 mm × 10 mm sample of the osteotome cutting edge was removed by wire spark-erosion. Each cutting-edge sample then underwent scanning electron microscopy SEM analysis and Energy-dispersive x-ray spectroscopy EDS (SEM/EDS, Hitachi SU-70) to detect surface damage and traces of biological contamination.

The sterilization process consisted in a multiple thermal cycling, placing the cleaned osteotomies in an autoclave at 121°C for 60 mins at 15 bar pressure. After which, they were subsequently quenched in chilled water to 5°C for 60 seconds and left to air dry. This thermo-cycling process was repeated four times following each of the four bone cutting cycles.

## Results

**Fig 1:**
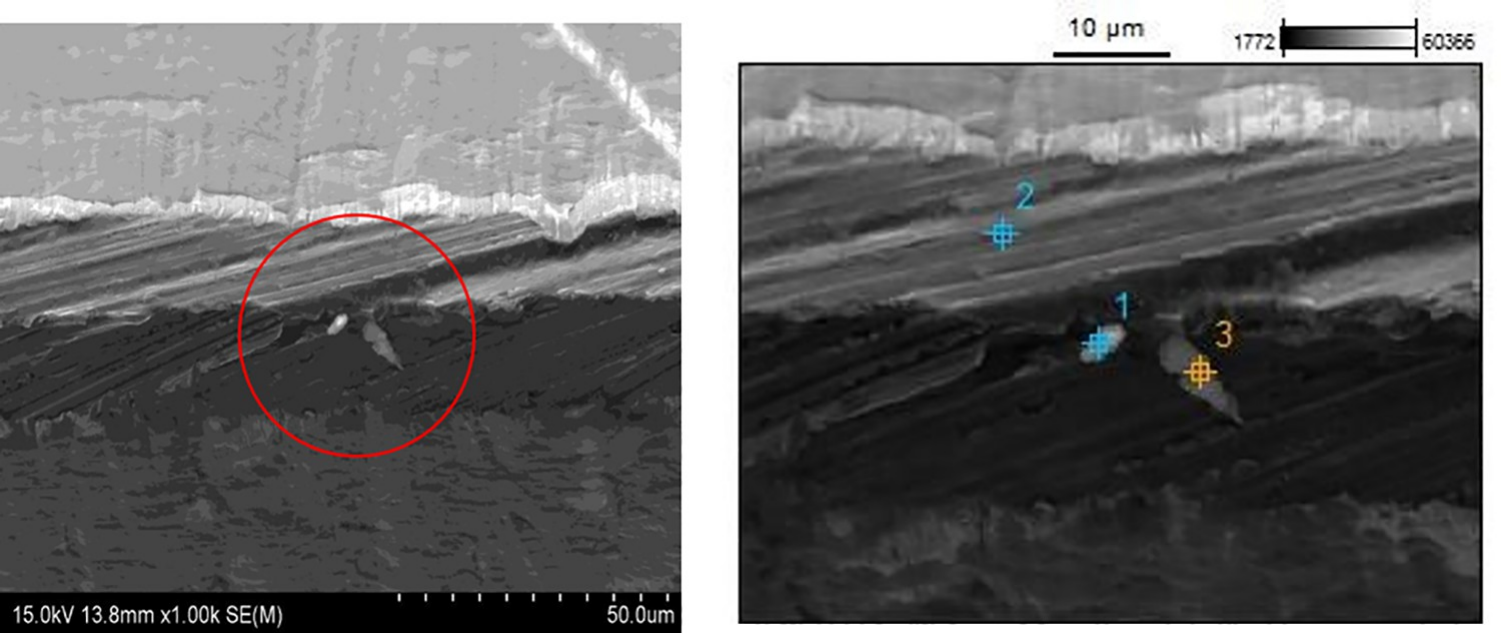
As-supplied osteotome cutting edge at x1000 magnification (left). EDS points for as-supplied osteotome (right). Mechanical wear and damage can be seen across cutting edge.

SEM image × 1000 magnification of the three osteotome blade samples identified different levels of wear damage forming on the cutting face however the worst wear was detected on the as-supplied osteotome (Fig 1 -left).

**Fig 2:**
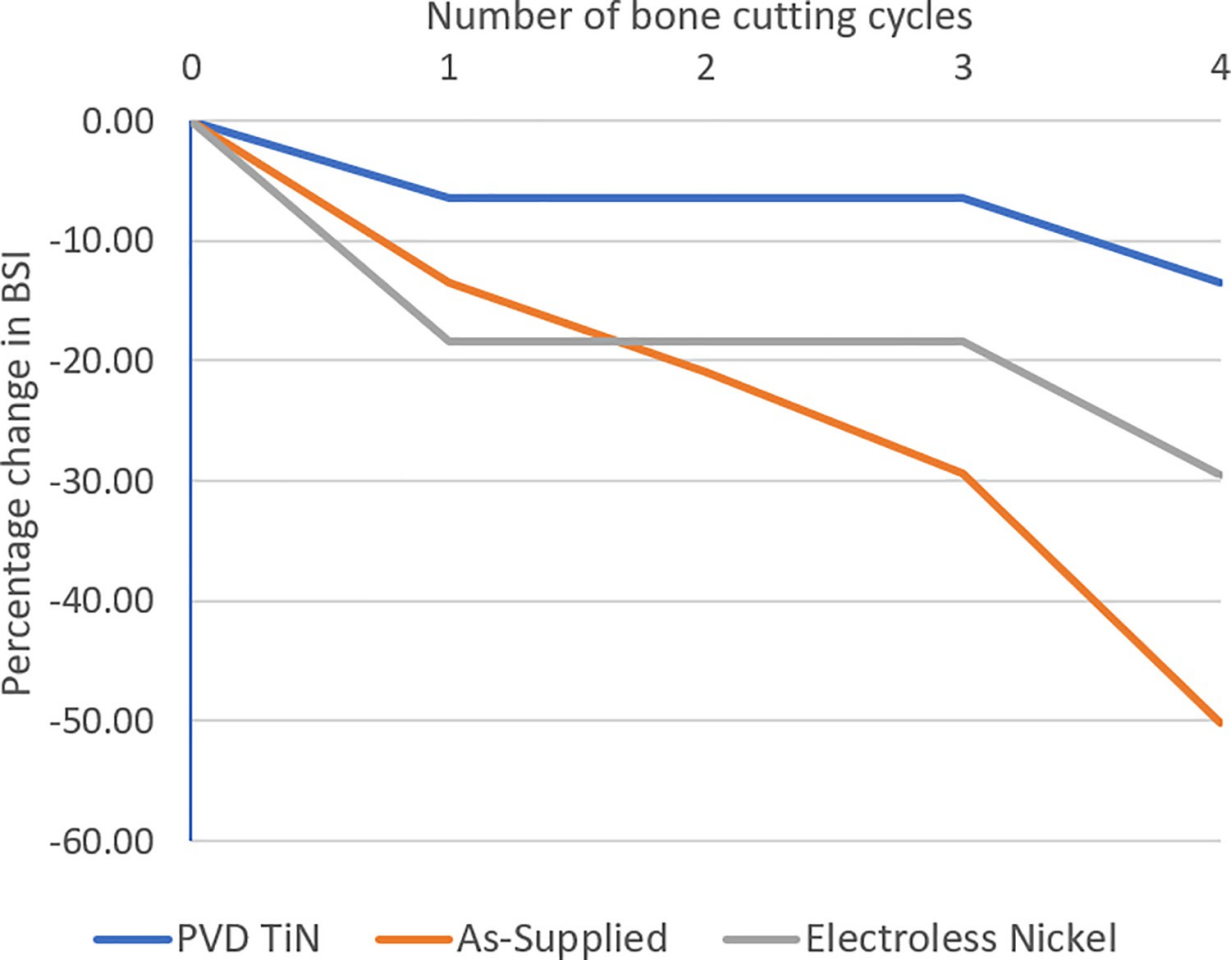
Degradation in osteotome blade sharpness index (BSI) measured over four bone cutting cycles, (0 = new).

Cutting edge degradation over four bone cutting cycles in all three osteotomes (Fig 2), using methods developed by McCarthy et al. [9], demonstrated a reduction in blade sharpness index (BSI) to differing degrees in all three osteotomes. Here the as-supplied osteotome experienced the most degradation in cutting edge sharpness, experiencing a 50% reduction in BSI, while the TiN coated osteotome experienced the least wear and damage, demonstrating a 13% reduction in BSI.

**Fig 3:**
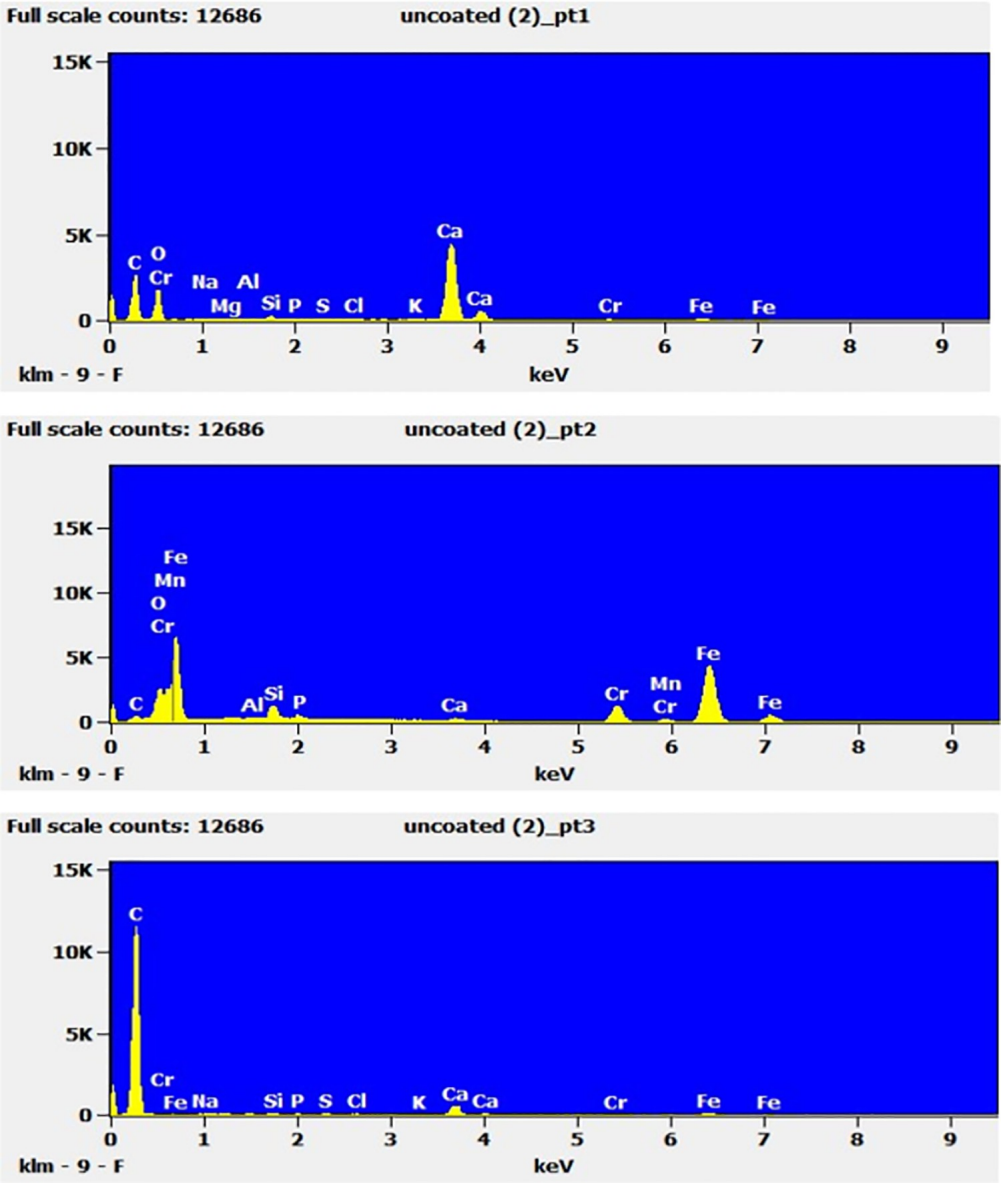
EDS Scan for Point 1 (top), Point 2 (middle) and Point 3 (bottom) for as supplied osteotome.

In all three osteotomes, EDS was performed on the encircled area in different selected points 1,2,3 (Fig 1 -right) to determine the material composition present on the damaged cutting surface. Unsurprisingly, the as-supplied osteotome that demonstrated the greatest reduction in BSI, correlating to the greatest cutting-edge wear and mechanical damage, also recorded the highest level of bone contamination. point 1 (Fig 3, top) presents a mixture of bone contaminants amongst the base material in the as-supplied osteotome after four bone cutting cycles. The larger reading of calcium, 5,000 units, and smaller readings of phosphorous, 100 units, are evidence of bone contaminants, while chromium, magnesium, silicon, aluminum, carbon, and nickel are from the base material of stainless steel. Loss in BSI and bone contamination is presented by Table 1.

**Table 1:** Relationship between loss of cutting-edge sharpness and bone contamination.

	Wear	Bone Contamination	
	BSI Reduction	Ca (peak units)	P (peak units)
**As-supplied**	**50%**	**5,000**	**150**
**Electroless nickel**	**30%**	**3,500**	**400**
**TiN**	**13%**	**100**	**50**

In all EDS testing, traces of chlorine were also observed which is attributed to the chlorine used in the autoclave sterilization water. Points 2 and 3 (Fig 3, middle and bottom) show similar readings as point 1 with a mixture of bone contaminants, base material, and chlorine.

While less mechanical wear and damage occurred on the cutting edge in both the PVD and electroless nickel coating osteotomies, as shown by Fig 2, a large amount of titanium and nitrogen was also present in the EDS scans from the PVD osteotome which was attributed to the composition of this coating. As expected, there was also high nickel levels reported in the electroless nickel coated osteotome.

Bone contaminants in the electroless nickel coated osteotome were also evident with the detection of calcium and phosphorus but at lower levels to that found in the as supplied osteotome. The PVD coated osteotome demonstrated significantly less bone contamination than either the as-supplied or electroless nickel coated samples.

## Discussion and conclusions

According to the results there is an association between blade sharpness and post-sterilization bone contamination. This relationship could be explained by loss of cutting edge causing both higher cutting forces and greater impact mechanical damage to the bone. This mechanical damage could also provide a site to harbour bone residue.

While testing has shown the PVD coated osteotome to have less bone contamination compared to the electroless nickel and as-supplied examples, it remains an issue that any amount of bone residue can lead to patient cross-contamination and potential source of post-surgical infection when the tool is reused.

Although this pilot study has its own limitations, and a broad sample analysis should be performed to determine to what extent biological material is present after sterilization process, and how this presence could be mitigated with a coating procedure. The results of this study demonstrate the degree of bone contamination is proportional to cutting-edge wear, and that these two phenomenon cannot be completely eliminated. This suggests either single-use disposable osteotomes should be used in the surgical procedures, or effective sharpening processes be undertaken between each operation to avoid infections or at least as a preventive measure.

Previous studies undertaken [10] had reported that over 290,000 surgical site infections occurred annually within the United States, including some that were fatal. These post-operative infections were reported to occur within 30 days to a year after surgery, depending on the type of procedure, resulting in high direct and indirect costs estimated to range from 1 billion to 10 billion US dollars. Therefore, cross contamination and surgical site infections can prove to be very expensive, occasionally fatal, and should be prevented as much as possible.

In conclusion, while hospitals currently have procedures to maintain and regularly sharpen osteotomies to minimize these issues, there is still a high risk of patient cross-contamination given the rate of rapid cutting-edge degradation that occurs. This mechanical degradation provides a harbour for contaminant material to reside. The results of our study suggest that PVD coating surgical cutting instruments may be effective in reducing these issues by providing a more durable cutting edge however this coating prevents re-sharpening. Single use disposable osteotomes may also be another potential solution.
